# Lanthanide-Doped Nanoparticles for Diagnostic Sensing

**DOI:** 10.3390/nano7120411

**Published:** 2017-11-23

**Authors:** Song Yeul Lee, Min Lin, Aeju Lee, Yong Il Park

**Affiliations:** 1 School of Chemical Engineering, Chonnam National University, Gwangju 61186, Korea; lord_c@naver.com; 2 The Key Laboratory of Biomedical Information Engineering, Ministry of Education, School of Life Science and Technology, Xi’an Jiaotong University, Xi’an 710049, China; minlin@xjtu.edu.cn; 3 Bioinspired Engineering and Biomechanics Center (BEBC), Xi’an Jiaotong University, Xi’an 710049, China; 4 International Research Organization for Advance Science and Technology (IROAST), Kumamoto University, Kumamoto 860-8555, Japan; aeju-lee@kumamoto-u.ac.jp; 5 Magnesium Research Center, Kumamoto University, Kumamoto 860-8555, Japan

**Keywords:** lanthanide, upconversion, nanoparticles, diagnostics, sensing

## Abstract

Lanthanide-doped nanoparticles exhibit unique optical properties, such as a long luminescence lifetime (up to several milliseconds), sharp emission peaks, and upconversion luminescence over the range of wavelengths from near-infrared to visible. Exploiting these optical properties, lanthanide-doped nanoparticles have been widely utilized for cellular and small animal imaging with the absence of background autofluorescence. In addition, these nanoparticles have advantages of high signal-to-noise ratio for highly sensitive and selective diagnostic detection. In this review, we summarize and discuss recent progress in the development of highly sensitive diagnostic methods using lanthanide-doped nanoparticles. Combined with a smartphone, portable luminescence detecting platforms could be widely applied in point-of-care tests.

## 1. Introduction

Disease diagnosis is the starting point of patient treatment, and fast and accurate diagnosis is highly demanded. Among the various diagnostic techniques, such as magnetic resonance imaging [1], computed tomography [2], and positron emission tomography [3], optical detection using a fluorescence signal has been widely used [4,5]. Fluorescence-based detection has intrinsic limitations, such as the shallow penetration of light [6] and inability to be applied for whole body imaging. In spite of these limitations, fluorescence-based diagnostic systems are useful for identifying disease targets in blood. Fluorescence detection allows rapid and accurate signal detection, and fluorescence detection instruments are abundant in laboratories and hospitals.

In fluorescence-based detection, various types of imaging probes have been commercialized. Most imaging probes are fluorescent organic dyes that are used for sensing target ions or molecules. However, organic dyes have several drawbacks, such as poor chemical stability, photo-bleaching, broad emission spectra, and difficulties in conjugation with targeting ligands. Thus, inorganic nanoparticles (NPs) have been developed to replace conventional fluorescent organic dyes [7]. Among them, semiconductor quantum dots (QDs) are promising candidates for fluorescence-based detection [8,9,10]. Compared with organic probes, QDs have advantages, such as good chemical and photo-stability, a tunable emission wavelength by controlling the particle size, easy conjugation with biomolecules, and narrow emission spectra for less spectral overlap. Over the last two decades, QDs have been applied in the fields of animal imaging and disease marker sensing [11,12]. Even though QDs exhibit interesting properties, their usage has been limited by critical issues such as potential human toxicity. Common QDs are composed of heavy metals (e.g., cadmium, lead, and mercury), which are potentially fatal to humans. In addition, there was a need to develop imaging probes with better detection sensitivity than QDs. To overcome these obstacles, new kinds of lanthanide-doped NPs have attracted attention as the next generation of imaging probes for highly sensitive fluorescence detection.

Owing to their narrow emission spectra and large Stokes and anti-Stokes shifting emission [13], lanthanide-doped NPs can detect the targets with high sensitively and selectively. Unlike QDs, the emission wavelength and bandwidth of lanthanide-doped NPs are determined by the atomic energy level of the lanthanide [14,15]. The emission wavelength is easily tunable by controlling the elemental composition, and the long luminescence lifetime of lanthanides enables the removal of background noise using time-resolved (TR) detection. Sharp emission peaks allow minimal spectral overlap for multicolor imaging [16,17]. In particular, when utilizing the anti-Stokes shifting process, lanthanide-doped NPs can convert the low energy photons into high energy photons; hence, they are called upconverting NPs (UCNPs) [18]. This means that near-infrared (NIR) wavelengths can be used as the excitation source, and visible light or even ultraviolet (UV) light can be observed. When using NIR excitation, photo-induced damage and the background signal are significantly reduced [19].

The weak luminescence intensity of lanthanide ions is not sufficient for highly sensitive detection. Therefore, to enhance the luminescence intensity, lanthanide ions are incorporated into host materials such as polymer beads [20,21], silica [22], and inorganic NPs [23]. Although bare NPs without any surface modification could be used for target detection [24], bioconjugation of NPs with target-specific moieties (e.g., proteins or nucleic acid) is commonly recommended to increase the sensitivity and specificity [25].

Very recently, portable luminescence-based detection systems have been developed for point-of-care tests [26]. Combined with a smartphone, portable luminescence detecting platforms could be widely applied in e.g., a road-side assay for the diagnosis of infectious disease at a critical location. In addition, owing to their unique and excellent optical properties (e.g., long luminescence lifetime, minimal background autofluorescence), lanthanide-doped NPs could contribute to the development of such portable diagnostic devices with high sensitivity and specificity [27]. In this review, we summarize and discuss the recent progress in the development of highly sensitive diagnostic methods using lanthanide-doped nanoparticles, which could contribute to the portable diagnostic system for the point-of-care test in the future.

## 2. Lanthanide-Doped Luminescent Nanoparticles for Diagnostic Sensors

Lanthanide ions have intrinsic luminescence and ladder-like energy states. They can absorb photons in the UV, visible, and even NIR regions depending on their specific energy levels [14,15]. They also have unique optical properties such as large Stokes or anti-Stokes shifts, sharp emission peaks, and long-lived luminescence. Moreover, lanthanide-doped inorganic NPs exhibit no photo-bleaching or photo-blinking [28,29]. Owing to these merits, lanthanide-doped NPs can be used as detecting probes or sensors. For example, lanthanide-doped NPs can be used for multi-target detection as they have narrow emission bands. In addition, the long-lived luminescence enables the use of a time-resolved detection technique [30,31] where the short-lived background signal can be eliminated and the sensitivity can be increased compared to conventional fluorescent sensors.

Lanthanide-doped NPs have been applied in the fields of both homogeneous and heterogeneous photoluminescence (PL) assays. The homogeneous PL assay uses a mixture of liquid-phase materials. This assay is considered as a convenient method for rapidly detecting targets through a simple “mix-and-read” step. The homogeneous PL assay usually works through the principle of energy transfer, which depends on the distance between the donor (generally lanthanide-doped NPs) and acceptor that usually acts as a quencher (e.g., carbon sheets [32], gold nanorods (GNRs) [33,34], gold NPs (AuNPs) [35], and fluorescent dyes [20]). Thus, the efficiency of this energy transfer system depends on the integral of the spectral overlap between the emission bands of the donor materials and the absorption bands of the acceptor materials as well as the distance between the donor and acceptor materials. Meanwhile, the heterogeneous PL assay refers to a solid-phase assay. This assay has several advantages, including a high specificity for target analytes and a high binding affinity between the target analytes and NPs. Typical heterogeneous PL assays are sandwich-type assays that involve target molecules being captured by a specific ligand on the substrate and then sandwiched by a reporter ligand [36,37], which generates the fluorescent signal through energy transfer. To capture targets, the surface of the NPs is usually modified with recognition moieties such as biotin, avidin, antibody, and aptamer.

### 2.1. Stokes Shifting Luminescent Nanoparticles

Lanthanide complexes are commonly used as biological probes [38,39]. Although the lanthanide ion itself has luminescent properties, the intensity is usually weak because of the forbidden nature of the f-f absorption transition. To overcome this limitation, lanthanide complexes are synthesized with attached chromophores. As the lanthanide ions have strong coordination with nitrogen and oxygen atoms, organic materials containing O and N are generally used as coordination ligands for preparing lanthanide complexes. A high signal-to-noise ratio is required for effective sensing of target molecules. Therefore, lanthanide-doped NPs based on polymer, silica, and sodium metal fluoride have been synthesized to increase emission intensity. In addition, TR detection is usually used to remove background noise originating from UV or visible excitation.

**Figure 1:**
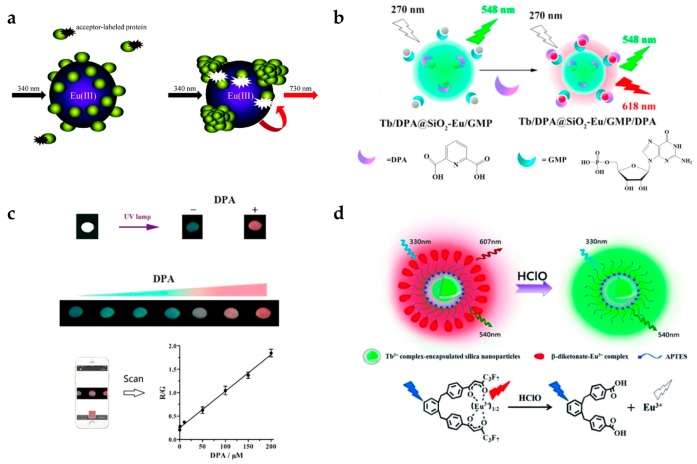
(**a**) Design concept of protein aggregation analysis using a luminescence resonance energy transfer (LRET) with Eu-doped polystyrene (PS) nanoparticles (NPs). Protein aggregation exposes the surface of PS NPs leading to the adsorption of dye-labeled protein. (**b**,**c**) The principle of dipicolinic acid (DPA) detection; (**b**) Surface binding of DPA induces the strong red emission of Eu/GMP by antenna effect; (**c**) Luminescence color images of DPA detection and ratiometric detection using dual-color channels; (**d**) Schematic illustration of hypochlorous acid (HClO) measurement. HClO induces ratiometric luminescence quenching by releasing Eu ions from Eu-complex. Reprinted with permission from [20,22,42].

Pihlasalo et al. designed a detection system for protein aggregation using Eu-doped polystyrene (PS) NPs (Figure 1a) [20]. Appropriate analytical methods for detecting and quantifying protein aggregation are important as protein aggregation is known to be related to neurodegenerative conditions (e.g., Alzheimer’s and Parkinson’s disease) [40] and prion disease [41]. The surface of the Eu-doped NPs was covered by non-aggregated proteins, and the adsorption of acceptor-labeled proteins (Alexa Fluor 680-labeled gammaglobulin) was blocked, inhibiting luminescence resonance energy transfer (LRET) from Eu to the acceptor. The LRET process is not efficient, owing to their long distance between Eu and Alexa Fluor 680. When protein aggregation on the nanoparticle was initiated, the uncovered surface was exposed and the acceptor-labeled protein adsorbed, resulting in LRET activation. By measuring the 730 nm emission from Alexa Fluor 680, the degree of protein aggregation could be quantified.

Zhang et al. designed a system for detecting an anthrax biomarker, dipicolinic acid (DPA), using lanthanide-doped silica NPs (Figure 1b) [22]. Some fluorescent probes have already been used to detect DPA. However, their sensitivity was limited owing to the unavoidable background noise. To overcome this limitation, the TR technique was adopted [43]. Dual-color lanthanide-doped silica NPs (Tb/DPA@SiO_2_-Eu/GMP) were used as the luminescent probe. The Tb/DPA complex inside silica emitted strong green light by the antenna effect of DPA, which was used as a reference signal. Eu/GMP on the silica surface binds with DPA, and red emission was also enhanced by the antenna effect. Therefore, the luminescence changed from green to red with increasing DPA concentration, and the detection limit was 7.3 nM (Figure 1c). NPs using dual-color channels could be used as ratiometric luminescence probes providing more accurate detection than single-channel detection. In addition, they investigated whether this assay had selectivity to the target molecule. It is clearly shown that only DPA resulted in an increase in the fluorescence ratio (F_618_/F_548_) among several aromatic ligands and amino acids. In addition, the color change from green to red was observable by the naked eye. The group of Yuan also reported dual-emission lanthanide-doped silica NPs using the TR method [42]. In particular, they focused on detecting hypochlorous acid (HClO) [44], which plays a critical role in the immune system. However, if there is an excess of HClO, it can cause tissue damage and some human diseases such as lung and cardiovascular diseases and certain cancers. For this reason, it is necessary to detect HClO with high selectivity and sensitivity. This group produced NPs composed of Tb complex-encapsulated silica NPs and a β-diketonate-Eu complex as a ratiometric TR luminescence probe (Figure 1d). The nanoprobe emitted in both green (540 nm) and red (607 nm) under 330 nm excitation. In the presence of HClO, Eu ions are released from β-diketonate, and red emission from Eu disappears. It was observed that the emission peaks at 607 nm (^5^D_0_ → ^7^F_5_ transition of Eu) decreased with increasing HClO concentration, while the emission peak at 539 nm from Tb had a constant intensity. By measuring the ratio of green to red emission, HClO could be measured with high selectivity and sensitivity.

The group of Chen reported lanthanide-doped CaF_2_ NPs for detection of a tumor marker, urokinase plasminogen activator receptor (uPAR) [23]. CaF_2_:Ce,Tb NPs were conjugated with the amino-terminal fragment (ATF) of urokinase plasminogen activator (uPA), which binds specifically to the target uPAR. The intensity of luminescence at 491 nm (from Tb) gradually decreased with increasing uPAR concentration. On the contrary, the intensity of emission at 520 nm (from fluorescein isothiocyanate (FITC), which acted as an acceptor) increased through the LRET mechanism.

**Figure 2:**
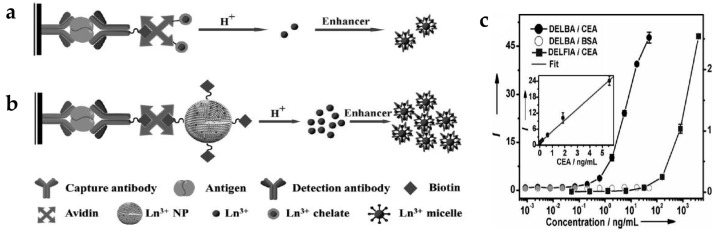
(**a**) Design concept of conventional dissociation-enhanced lanthanide fluoroimmunoassay (DELFIA) using lanthanide-chelates; (**b**) Schematic illustration of dissolution-enhanced luminescent bioassays (DELBA) using lanthanide NPs; (**c**) Titration data for the carcinoembryonic antigen (CEA) detection using DELFIA and DELBA. Reprinted with permission from [49].

Dissociation-enhanced lanthanide fluoroimmunoassay (DELFIA) (Figure 2a) is one of the most sensitive luminescent bioassay techniques and has been widely adopted in a variety of biomedical institutions [45,46]. There are several challenges when using conventional DELFIA. Lanthanide-chelates suffer from a low labeling ratio, thus providing limited time-resolved photoluminescence (TRPL) signal and sensitivity. In addition, the high cost of lanthanides and chelating agents and their weak chemical stability limit their practical application [47]. To solve these problems, inorganic lanthanide NPs have been applied to dissolution-enhanced luminescent bioassays (DELBA) [48]. Recently, the group of Chen introduced NaEuF_4_ NPs for DELBA (Figure 2b) [49], which showed many advantages: (1) NPs are more flexible for bioconjugation; (2) lanthanide nanocrystals are less toxic than lanthanide ions; (3) their cost is considerably lower than other materials. More importantly, they used an enhancer solution, 2-naphtoyltrifluoroacetone (β-NTA), to improve the luminescence intensity. When using this enhancer solution, the emission from the NPs was about 10^6^ times stronger than that of the NPs in phosphate-buffered saline (PBS). Therefore, strong emission of inorganic NPs in the enhancer solution could detect a carcinoembryonic antigen (CEA), an important tumor marker. As the concentration of CEA increased, the intensity of the TRPL signal increased accordingly; the limit-of-detection (LOD) was approximately 0.1 pg/mL (0.5 fM) in DELBA. This value is three orders of magnitude lower than that of the conventional DELFIA (90 pg/mL) [50], implying better detection sensitivity of DELBA (Figure 2c).

**Figure 3:**
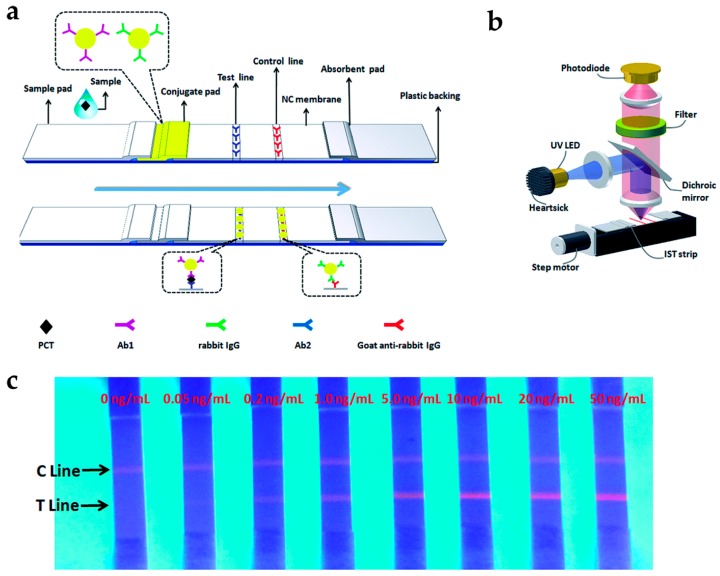
(**a**) Schematic representation of immunochromatographic strip tests (IST) for procalcitonin (PCT) detection; (**b**) Schematic of IST reader; (**c**) Quantitative analysis of the PCT levels using IST. Reprinted with permission from [27].

Li et al. developed a rapid and quantitative assay for procalcitonin (PCT), which is an early marker of bloodstream infection, using lanthanide-doped PS NPs (Figure 3) [27]. Eu-doped PS NPs were applied to immunochromatographic strip tests (IST), consisting of chromatography with a conventional immunoassay [51,52]. This test is a fast, simple, and low-cost analytical method. Eu-doped PS NPs were prepared by encapsulating the Eu-chelates into PS NPs which were modified with antibodies. The fluorescence intensity of Eu-doped PS NPs gradually increased as the PCT levels increased, and they have LOD as low as 0.05 ng/mL. The test in human serum took only 15 min to complete the sample analysis, which was eight-fold less than the commercial ELISA (~120 min). These results clearly demonstrate that the developed assay is a promising detection tool for PCT point-of-care detection.

The lanthanide complexes have been used as a common biological fluorescent tag, and commercial equipment for signal detection is readily available in laboratories and hospitals. Because the lanthanide-doped NPs using Stokes shift have similar fluorescent properties to lanthanide complexes, the NPs-based diagnostic assays can be easily transplanted to real applications using the related equipment. As shown above, the NPs have strong emission that results in high detection sensitivity. The long luminescence lifetime of NPs and TR imaging technique allows highly sensitive target detection without background noise interference. However, there are still some issues that need to be addressed. Since Stokes shifting NPs use UV as an excitation source, the sensitivity is limited by the background autofluorescence as in the case of QDs. The TR imaging also removes some of the emitted light from the probes while eliminating background signals, resulting in reduced signal intensity. Repeated measurements and signal attenuation can be applied to overcome these limitations. It is also necessary to develop novel NPs with strong emission.

### 2.2. Anti-Stokes Shifting Luminescent Nanoparticles

UCNPs are anti-Stokes emitting materials which can emit visible and even UV light by absorbing multiple NIR photons. As they use NIR light as an excitation source, background autofluorescence and photo-induced damage can be minimized. Minimal autofluorescence enables more sensitive luminescent detection with a high signal-to-noise ratio. Low photo-toxicity allows real-time imaging of live targets and their long-term monitoring. Hence, UCNPs are promising materials for biomedical applications, such as imaging probes or sensors.

**Figure 4:**
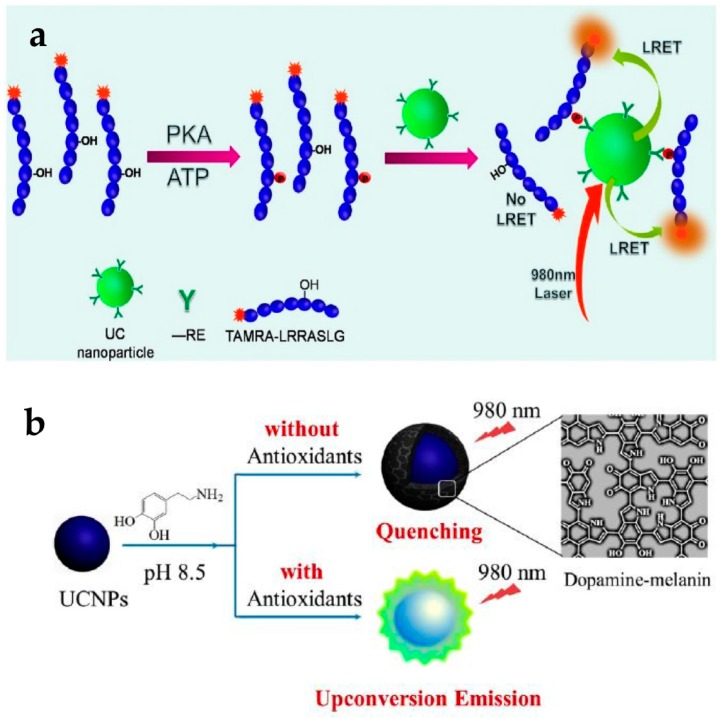
(**a**) Principle of protein kinase A (PKA) detection based on upconverting NP (UCNP) LRET assay; (**b**) Schematic representation of antioxidant assay based on UCNPs with dopamine. Reprinted with permission from [24,53].

Bare UCNPs could be used to detect protein kinases (PKs) without any targeting ligands. Dysfunction of some PKs can induce various human diseases including cancers; hence, it is highly desirable to detect the activity of specific PKs with high sensitivity using a simple method. UCNPs have been applied to detect tetramethylrhodamine (TAMRA)-labeled protein kinase A (PKA) specific peptide using a LRET system (Figure 4a) [24]. The UCNPs could intrinsically capture the TAMRA-labeled fluorescent phosphopeptides using the binding affinity between lanthanide ions on the particles and phosphate on the peptides. In this LRET system, UCNPs acted as the donor and TAMRA acted the acceptor. When the concentration of PKA increased, the emission intensity at 582 nm increased according to the energy transfer from UCNPs to TAMRA. This means that the high PKA activity induced more phosphorylated TAMRA-peptides and increased the LRET-based intensity. This UCNP-based assay could be employed for quantitative assessment of kinase inhibitors. Emission from TAMRA decreased with increasing H-89 concentration as this molecule acts as an inhibitor of PKA activity.

The use of UCNPs with dopamine could detect antioxidants such as biothiols, vitamin C, and Trolox. Dopamine easily polymerizes depending on the pH [54], and the resulting dopamine-melanin molecule acts as a luminescence quencher. The group of Ren reported the detection of various antioxidants using dopamine polymerization of UCNPs (Figure 4b) [53]. At a pH of 8.5 without antioxidants, the oxidative polymerization of dopamine resulted in the formation of a dopamine-melanin shell on the UCNPs, which quenched upconversion fluorescence. In the presence of antioxidants, oxidative polymerization is inhibited, resulting in no fluorescence quenching. It was shown that the PL intensity of UCNPs gradually increased with increasing antioxidant concentration. They also evaluated the specificity of a UCNP-dopamine hybrid system for antioxidants. Among the various compounds tested, only antioxidants showed the anti-quenching effect.

Although the UCNPs could be used to detect PKA and antioxidants without surface modification, conjugation of UCNPs with targeting ligands is required to achieve high sensitivity and specificity. Various types of targeting ligands, such as proteins (e.g., antibody, avidin) and oligonucleotides (e.g., DNA, RNA, aptamer), have been applied to the UCNPs. Zhang et al. used DNA-labeled UCNPs to detect methicillin-resistant *Staphylococcus aureus* (MRSA), which has resistance to general antibiotics [55]. UCNPs modified by capturing DNA exhibited high selectivity and sensitivity to the target MRSA DNA sequence. After capturing the target DNA, TAMRA-labeled reporter DNA was hybridized with the remaining target DNA, forming a LRET system. Higher MRSA concentrations resulted in a higher degree of LRET occurring between the UCNPs and TAMRA. Thus, as the concentration of MRSA increased, the luminescent intensity at 543 nm from the UCNPs decreased, while the emission intensity at 580 nm from TAMRA increases.

**Figure 5:**
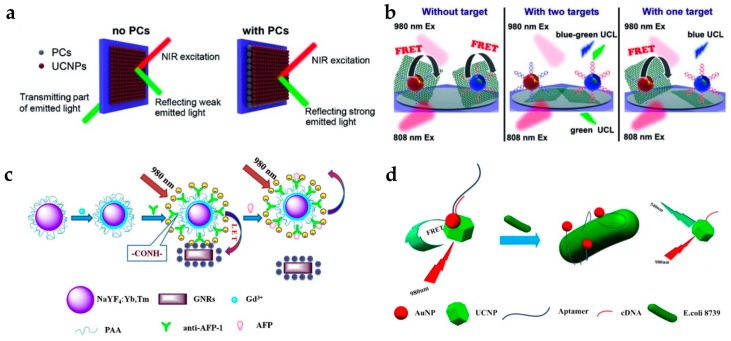
(**a**) Schematic of optical pathway when UCNPs are deposited on the PCs; (**b**) The principle of simultaneous detection of multiple targets; (**c**) Schematic illustration of α-fetoprotein (AFP) detection using gold nanorods (GNRs) as a quencher; (**d**) Schematic representation of bacteria detection using AuNPs as a quencher. Reprinted with permission from [32,33,35].

Yuan et al. studied the detection of mRNA, which is known to be an important disease marker for tumor growth [32]. They enhanced the detection efficiency of the upconversion signal using a photonic crystal (PC). The PC layer blocked transmitting light and increased the reflected fluorescent signal toward the detector, resulting in significantly enhanced upconversion intensity (Figure 5a). They also used graphene oxides (GO) as a luminescence quencher. Oligonucleotide-labeled UCNPs are easily attached to GO by the π-π interaction, and target mRNA hybridization on the UCNPs induced the detachment of UCNPs from GO (Figure 5b). In addition, two different types of UCNPs, NaYF_4_:Yb,Tm for blue emission under 980 nm irradiation and NaYF_4_:Yb,Er@Nd-doped shell for green emission under 808 nm irradiation, were used. These two UCNPs enabled the simultaneous detection of different targets as the blue and green emissions of UCNPs exhibited little overlap with each other. They detected C-myc mRNA and TK1-mRNA with a LOD of 0.01 nM.

The Chen group reported lanthanide-doped LiLuF_4_ for detection of the pregnancy and tumor marker, β subunit of human chorionic gonadotropin (β-hCG) [56]. They synthesized LiLuF_4_:Yb,Er conjugated with avidin. Avidin has been widely used for biotin binding. In this assay, the β-hCG antigen was captured by the biotinylated β-hCG antibody, and the avidin-labeled UCNPs were bound. It was clearly observed that higher concentrations of β-hCG resulted in stronger upconversion intensity.

Wang et al. developed a detection assay for tumor markers such as the α-fetoprotein (AFP) antigen and prostate-specific antigen (PSA) (Figure 5c) [33,34]. AFP is a tumor marker for hepatocellular carcinoma and yolk sac tumor, and PSA is a tumor marker for early detection of prostate cancer. In their studies, GNRs, which have strong absorption near 800 nm, were used as a quencher for the 800 nm emission from Tm-doped UCNPs. For AFP antigen detection, they designed a sensor with carboxyl-functionalized UCNPs conjugated with anti-AFP. Electrostatic interaction between the UCNPs-anti-AFP (negative surface charge) and GNRs (positive surface charge) bring them into close proximity, inducing luminescence quenching by energy transfer. The addition of the AFP antigen interrupted the energy transfer between the UCNPs and GNRs, resulting in the luminescence being recovered. The AFP antigen was measured over the detection range of 0.18–11.44 ng/mL, while the detection limit was 0.16 ng/mL. For PSA detection, they utilized a different approach; both the UCNPs and GNRs were conjugated with antibodies, anti-PSA-1 and anti-PSA-2. In the presence of the target PSA, the UCNPs and GNRs were bonded via a specific interaction between the antibody and antigen, resulting in luminescence quenching. Therefore, the quenching effect increased when the concentration of the target PSA increased. In addition, their sensor showed good selectivity, as the antibody was selectively bound to the specific antigen.

Kim et al. designed a sensor to detect glycated hemoglobin (HbA1c) [25]. The surface of the UCNPs was functionalized with the antibody of HbA1c and thus these particles could capture the target HbA1c. HbA1c can act as an acceptor and the energy transfer occurred from the UCNPs to the HbA1c. As a result, the capture of HbA1c quenched the luminescence from the UCNPs depending on their abundance.

As a prospective substitute for the antibody, the aptamer is used as a target-binding ligand with high binding affinity. Aptamers have several advantages: (1) they can selectively bind various targets from ions to cells; (2) easy synthesis and modification; (3) stable over a range of temperatures and pH values. Owing to these advantages, there are many recent reports using aptamers to detect various targets, such as *Escherichia coli* (*E. coli*), microcystin-LR (MC-LR), and thrombin [35,57,58]. The group of Lin reported the detection of *E. coli* 8739 using LRET between UCNPs and AuNPs (Figure 5d) [35]. As the AuNPs have absorption overlap with the green emission of UCNPs, AuNPs act as a luminescence quencher (or acceptor). In this system, UCNP-cDNA hybridized with AuNP-aptamer, green emission of the UCNPs was quenched. When E. coli was present, the affinity between the bacteria and AuNP-aptamer was stronger than that between the AuNP-aptamer and UCNP-cDNA. Therefore, the upconversion luminescence was recovered as the UCNPs were released from the AuNPs.

Wang et al. developed a sensor to detect MC-LR (a microcystin which acts as a toxic element in water supplies) using MoS_2_ nanosheets as a luminescence quencher [57]. Similar to GO, UCNPs-aptamer are easily attached on the MoS_2_ nanosheet by π-π interaction. The addition of the target MC-LR to the UCNP-MoS_2_ hybrid induced a change in the aptamer structure, and UCNPs were detached from the MoS_2_ nanosheet. The recovered luminescence increased depending on the concentration of the targets.

**Figure 6:**
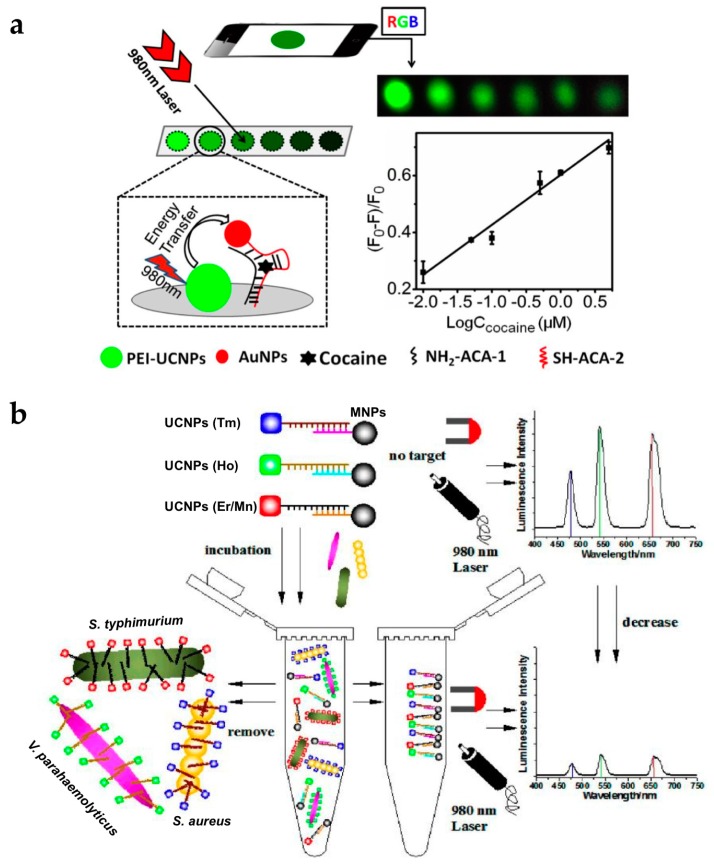
(**a**) Schematic of the UCNP-based paper assay for cocaine testing; (**b**) Schematic illustration of multiplexed luminescence assay for simultaneous detection of various bacteria. Reprinted with permission from [62,63].

To expand the application of UCNPs to roadside assays or point-of-care tests, it is essential to achieve sensing systems with portability and high selectivity and sensitivity. Roadside field testing is required globally as drug abuse is a serious social and health problem. For this purpose, the paper-based assay is attractive due to its simple and rapid operation, relatively cheap cost, and easy surface modification [59,60,61]. Recently, the group of Liu developed fast and simple paper-based cocaine testing using UCNPs, AuNPs, and the anti-cocaine aptamer (ACA) (Figure 6a) [62]. To construct the LRET system, the ACA was cut into two ssDNA pieces (ACA-1, and ACA-2). UCNPs, ACA-1, and AuNPs-ACA-2 were immobilized together on the cellulose filter. In the absence of cocaine, the LRET from the UCNPs to the AuNPs was not efficient and upconversion luminescence was constant. In the presence of cocaine, the aptamers formed tertiary structures, and the distance between the UCNPs and AuNPs decreased, resulting in luminescence quenching. Owing to the advantage of upconversion luminescence (e.g., minimal background noise and high signal-to-noise ratio), the change in luminescence could be measured using a smartphone or even detected by the naked eye.

In addition, there exists a report of the detection of an oligonucleotide of *E. coli* using a paper-based sandwich format [64]. This bioassay also used LRET and consisted of UCNPs as the energy donor and Cy3 dye as the energy acceptor. Oligonucleotide-labeled UCNPs on the paper captured the target DNA, and then reporter DNA with Cy3 hybridized the remaining target DNA. Formation of UCNP-Cy3 resulted in LRET from the UCNPs to Cy3, and the LRET ratio increased with increasing amount of target DNA.

The sharp emission spectra of UCNPs allow simultaneous detection of multiple targets [17]. The group of Wang reported the detection of various pathogenic bacteria using aptamer-modified UCNPs with different colors (Figure 6b) [63]. UCNP-aptamer and magnetic NP-cDNA (MNP-cDNA) structures were initially hybridized. In the presence of bacteria, the UCNP-aptamer particles were detached from the MNP-cDNA and bound to the target pathogen. Unbound UCNPs were easily retrieved by MNPs [65,66] and measured by the spectrometer. The emission peaks of the upconversion luminescence of *Staphylococcus aureus*, *Vibrio parahaemolyticus*, and *Salmonella typhimurium* did not overlap, which were observed at 477, 542, and 660 nm, respectively. In addition, it was clearly shown that the intensity of each wavelength gradually decreased with increasing concentration of the target bacteria. Therefore, the type of bacteria and their concentration could be simultaneously detected with high sensitivity and specificity.

Recently, Yüce et al. also designed an aptasensor to identify food pathogens [67]. They used two fluorescent materials, QDs and UCNPs, to separately detect *Staphylococcus aureus* and *Salmonella typhimurium*. Since QDs follow the Stokes shift and UCNPs follow the anti-Stokes shift, disturbance by same excitation source can be avoided. Emissions were recorded at 325 nm for QDs and 980 nm for UCNPs. This system efficiently detected the target DNA from different pathogens with only minor overlapping of the emission signals.

The use of NIR as an excitation source allows highly sensitive target imaging without background autofluorescence. The NIR LED is commercially available at high power, especially at 980 nm wavelength. Therefore, the UCNPs-based assay can give high sensitivity to the portable diagnostic device. However, there are still some issues that need to be addressed for UCNPs-based detection. Since the quantum yield of UCNPs is relatively low compared with Stokes shifting NPs, the brightness of the upconversion emission must be enhanced for sensitive detection. The sharp emission peak of UCNPs allows simultaneous detection of multiple targets with minimal spectral overlap. However, typical UCNPs exhibit multiple emission peaks from ladder-like energy states of lanthanides, and it is essential that the emission peaks from some types of UCNPs are decoded for multiplexing [17].

## 3. Conclusions

Lanthanide-doped NPs, which have superior luminescence properties compared to other materials, entered the diagnosis field as a new class of imaging probes. Their intrinsic long luminescence lifetime allows high-sensitivity TR detection. As UCNPs are activated by NIR sources, autofluorescence from the sample and photo-induced damage can be avoided. Owing to the sharp emission peaks, the spectral overlap is minimized and the lanthanide-doped NPs is applicable to simultaneous detection of multiple targets. The modification of these nanoparticles with specific biomolecules enhances detection sensitivity and selectivity. The lanthanide-doped NPs with high signal-to-noise ratio have the potential for practical applications such as point-of-care diagnostics. In addition, UCNPs exhibit upconversion luminescence response to a variety of external stimuli, and thus they can be applied to sensors that detect pH, electrical fields, magnetic fields, and temperature changes [68].

Nevertheless, many obstacles, such as the high cost of lanthanide elements and intrinsically low quantum yield of the UCNPs, still need to be solved before future commercialization is practical. Various strategies have been attempted to increase the quantum yield of the UCNPs, including surface passivation using core@shell structure and control of dopant distribution. Increasing the absorption cross-section in the NIR range is also necessary to improve the absorption efficiency of NIR photons. NIR dyes and gold nanostructures have been applied to UCNPs as antenna materials to aid NIR light absorption. These antenna materials can also adjust the range of absorption wavelength, allowing the flexible excitation sources to be selected. Multiple emission peaks of the lanthanide emitters can limit the practical multiplex detection. Although there are several reports of the single band red emission of UCNPs, emission wavelength tuning for single band emission is still a challenging issue. In addition, studies on the surface modification for functionalization and colloidal stability is needed for practical application in real world. Therefore, it will require the extensive collaboration of researchers in various fields, such as chemistry, engineering, and biology.

## Abbreviations

ACA	anti-cocaine aptamer
AFP	α-fetoprotein
ATF	amino-terminal fragment
AuNPs	gold nanoparticles
β-hCG	β subunit of human chorionic gonadotropin
β-NTA	2-naphtoyltrifluoroacetone
CEA	carcinoembryonic antigen
DELBA	dissolution-enhanced luminescent bioassays
DELFIA	dissociation-enhanced lanthanide fluoroimmunoassay
DPA	dipicolinic acid
FITC	fluorescein isothiocyanate
GNRs	gold nanorods
GO	graphene oxides
HbA1c	glycated hemoglobin
HClO	hypochlorous acid
IST	immunochromatographic strip tests
LED	light emitting diode
LOD	limit-of-detection
LRET	luminescence resonance energy transfer
MC-LR	microcystin-LR
MNP	magnetic nanoparticle
MRSA	methicillin-resistant *Staphylococcus aureus*
NIR	near-infrared
NPs	nanoparticles
PBS	phosphate-buffered saline
PC	photonic crystal
PCT	procalcitonin
PKA	protein kinase A
PKs	protein kinases
PL	photoluminescence
PS	polystyrene
PSA	prostate-specific antigen
QDs	quantum dots
TAMRA	tetramethylrhodamine
TR	time-resolved
TRPL	time-resolved photoluminescence
UCNPs	upconverting nanoparticles
uPA	urokinase plasminogen activator
uPAR	urokinase plasminogen activator receptor
UV	ultraviolet
